# Synthesis process optimization and field trials of insecticide candidate NKY-312

**DOI:** 10.1038/s41598-021-86475-w

**Published:** 2021-03-25

**Authors:** Haiqi Wang, Hongjian Song

**Affiliations:** 1 State Key Laboratory of Elemento-Organic Chemistry, College of Chemistry, Nankai University, Tianjin, 300071 People’s Republic of China; 2 Department of Chemistry, School of Science, Tianjin University, Tianjin, 300072 People’s Republic of China

**Keywords:** Catalysis, Organic chemistry

## Abstract

NKY-312 is a highly active insecticide candidate with a simple structure. In order to carry out field trials and toxicity tests, its scale preparation is urgently needed, but the final step of the original synthetic route is a low-yielding sulfonylation reaction that generates a high proportion of a bissulfonylated by-product, its foliar contact activities against bean aphid (80% at 100 mg/kg) is significantly lower than that of NKY-312 (100% at 5 mg/kg), and uses pyridine as the solvent. In this work, we developed a highly selective (4-dimethylaminopyridine)-catalyzed monosulfonylation reaction that avoids the use of pyridine as a solvent and shows a much higher yield (98% yield with 98% HPLC purity) than the original reaction (68%). Then, we carried out the field trials and toxicity tests. In field experiments, the activities of NKY-312 against rice planthopper and wheat aphid were equal to pymetrozine and imidacloprid respectively.

## Introduction

**Figure 1:**
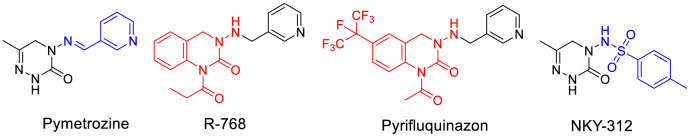
Chemical structures of pymetrozine, R-768, pyrifluquinazon, and insecticideal candidate NKY-312.

Phytophagous aphids can cause considerable damage to agricultural and horticultural plants^1^. Aphids suck juice out of plant tissues through their oral needles, which retards plant growth; in addition, they can spread plant viruses, which actually cause more damage to crops than the aphids themselves^2^. Insecticidal chemicals are currently the main tool used for controlling aphids. Pymetrozine, a pyridine azomethine compound that was developed by Syngenta in the 1990s^3^, as a chordotonal organ TRPV channel modulators, shows excellent activity against homopteran insects, especially aphids, whiteflies, and black tail leafhoppers. In addition, it has long-lasting efficacy and low mammalian toxicity, and organophosphorus and carbamate insecticides do not confer cross-resistance to it. Attempts to develop related compounds by modifying the structure of pymetrozine have been the focus of considerable research^4–14^. However, only two candidates have been generated to date, R-768^15^ and pyrifluquinazon^16^, both which are far less effective than pymetrozine (Fig. 1).

**Figure 2:**
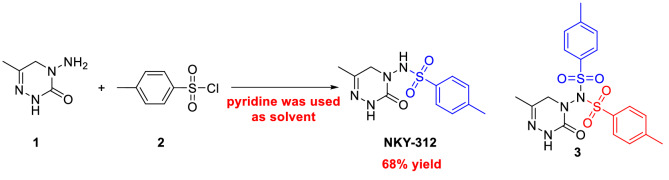
Previously reported synthesis of insecticide candidate NKY-312.

Because sulfonyl compounds have been reported to display insecticidal^17,18^, fungicidal^19^, herbicidal^20^, and antitumor^21,22^ activities, we previously designed and synthesized a series of triazinone sulfonamide derivatives of pymetrozine. In a greenhouse assay, one of these compounds, NKY-312 (Fig. 1)^23–26^, showed higher insecticidal activity against bean aphid than pymetrozine at 5 mg/kg (100% versus 30%). The penultimate intermediate in the industrial route to NKY-312 is 4-amino-6-methyl-4,5-dihydro-1,2,4-triazin-3(2*H*)-one^27^, which is subjected to a sulfonylation reaction to afford NKY-312 (Fig. 2). This final step has many disadvantages^23–26^ including the generation of a bissulfonylated by-product **3** that is difficult to separate from the desired product; the use of pyridine as a solvent; a low yield (68%); and unsuitability for scale-up. Meanwhile, the foliar contact activities against bean aphid of by-product (80% at 100 mg/kg) is significantly lower than that of NKY-312 (100% at 5 mg/kg)^23–26^. In order to carry out field trials and toxicity tests, large amount sample is needed; the development of a new, clean process for selective monosulfonylation is urgently needed.

**Figure 3:**
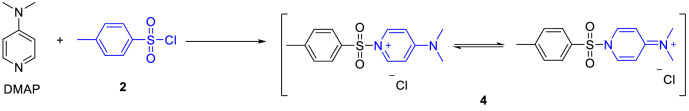
*N*-Toluenesulfonyl-4-dimethylaminopyridine salt.

We speculated that 4-dimethylaminopyridine (DMAP) might be useful for this purpose^28^. DMAP reacts with acylation reagents to form *N*-acyl-4-dimethylaminopyridine salts^29^, which exhibit charge delocalization that leads to the formation of compact ion pairs (Fig. 3). As a result, these salts react with nucleophiles as a unit. In this work, we developed a highly selective (4-dimethylaminopyridine)-catalyzed monosulfonylation reaction that avoids the use of pyridine as a solvent and shows a much higher yield than the original reaction.

## Results and discussion

### Screening of solvent, base, and reaction temperature

**Figure 4:**
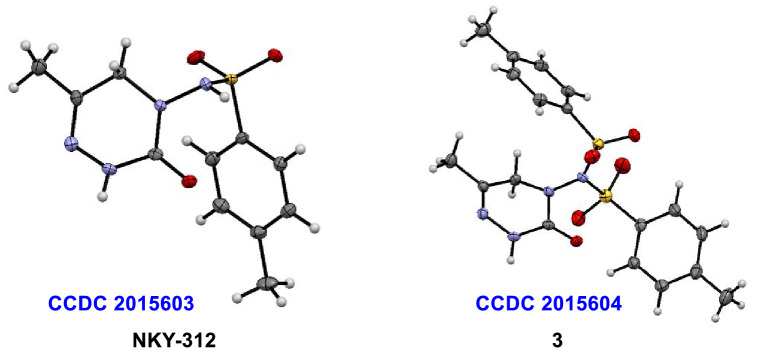
X-ray structures of NKY-312 and the bissulfonylated by-product **3.**

In the previously reported sulfonylation step, pyridine is used as both the base and the solvent. Here, we reduced the amount of pyridine to 1.5 equiv, used 10 mol % DMAP as the catalyst, and screened several solvents (dichloromethane [DCM], dimethyformamide [DMF], methyl-2-pyrrolidinone [NMP], dioxane, and tetrahydrofuran [THF]; Table 1, entries 1–5). We found that the reaction of **1** and *p*-toluenesulfonyl chloride (**2**) in DCM gave the highest yield of the desired monosulfonylation product (58%, entry 1), and the NKY-312:**3** ratio was > 50:1. Using DCM as the solvent, we then screened various organic and inorganic bases (entries 6–10), but no improvements were observed. Specifically, reactions with K_2_CO_3_, Na_2_CO_3_, and triethylamine as the base gave mainly the bissulfonylated by-product, the structure of which was confirmed by single crystal X-ray analysis (Fig. 4)^30^. Then we varied the reaction temperature. When both the addition of the sulfonyl chloride and the subsequent reaction were carried out at 0 °C, the solubility of the raw materials was poor, and the reaction time had to be extended to 18 h (entry 11). When both the sulfonyl chloride addition and the subsequent reaction were carried out at room temperature, the target product was obtained in 51% yield (entry 12).

**Table 1:** Screening of solvent, base, and reaction temperature^a^.


Entry	Base	Solvent	T (°C)	Yield (%)^b^	NKY-312:**3**
1	Pyridine	DCM	0 → rt	58	> 50:1
2	Pyridine	DMF	0 → rt	23	> 50:1
3	Pyridine	NMP	0 → rt	27	> 50:1
4	Pyridine	Dioxane	0 → rt	18	> 50:1
5	Pyridine	THF	0 → rt	40	> 50:1
6	K_2_CO_3_	DCM	0 → rt	< 5	1:21
7	Na_2_CO_3_	DCM	0 → rt	< 5	1:5
8	NaOH	DCM	0 → rt	Trace	–
9	Triethylamine	DCM	0 → rt	< 5	1:4
10	DBU	DCM	0 → rt	N.R	–
11^c^	Pyridine	DCM	0	49	> 50:1
12	Pyridine	DCM	rt	51	> 50:1

^a^Reaction conditions, unless otherwise noted: *p*-toluenesulfonyl chloride (**2**, 5.25 mmol) was added dropwise over the course of 10 min to a solution of **1** (5 mmol), DMAP (10 mol %), and base (1.5 equiv) in solvent (20 mL) at 0 °C, and the reaction was then allowed to proceed for 10 h at room temperature. DBU = 1,8-diazabicyclo[5.4.0]undec-7-ene. ^b^Yields were determined by HPLC (external standard method). N.R. = no reaction. ^c^The reaction time was18 h.

### Screening of amounts of dmap, pyridine, solvent, and 2

Having optimized the solvent, base, and temperature, we explored various other reaction parameters (Table 2). First, we varied the amount of DMAP (entries 1–4) and found that the highest yield was obtained with 1 mol % DMAP (entry 3). Next we screened various concentrations of **1** (entries 3 and 5–8). Increasing the concentration turned out to be beneficial: the yield of NKY-312 was highest (89%) when the concentration of **1** was 0.5 mol/L (entry 5). Then we varied the amount of sulfonyl chloride **2** (entries 5 and 9–11) and found that increasing the amount was deleterious; it is possible that the excess sulfonyl chloride increased formation of the bissulfonylated by-product, leading to a corresponding decrease in the yield of NKY-312. Finally, we screened the amount of pyridine (entries 5 and 12–14) and found that 2.5 equiv was optimal, affording NKY-312 in 98% yield (entry 13).

**Table 2:** Screening of Amounts of DMAP, Pyridine, Solvent, and **2**^a^**.**


Entry	DMAP (mol %)	Pyridine (equiv)	Concn (mol/L)	**2** (equiv)	Yield (%)^b^
1	15	1.5	0.25	1.05	50
2	5	1.5	0.25	1.05	58
3	1	1.5	0.25	1.05	78
4	0.5	1.5	0.25	1.05	60
5	1	1.5	0.5	1.05	89
6	1	1.5	0.16	1.05	76
7	1	1.5	0.125	1.05	72
8	1	1.5	0.6	1.05	85
9	1	1.5	0.5	1.1	89
10	1	1.5	0.5	1.15	84
11	1	1.5	0.5	1.25	83
12	1	2	0.5	1.05	94
13	1	2.5	0.5	1.05	98
14	1	3.5	0.5	1.05	98

^a^Reaction conditions, unless otherwise noted: *p*-toluenesulfonyl chloride (**2**, 1.05 equiv) was added dropwise over the course of 10 min to a solution of **1** (5 mmol), DMAP (1 mol %), and pyridine (2.5 equiv) in DCM (10 mL) at rt, and the reaction was allowed to proceed for 10 h at rt. ^b^Yields were determined by HPLC (external standard method).

### Scaled-up monosulfonylation reaction

**Figure 5:**
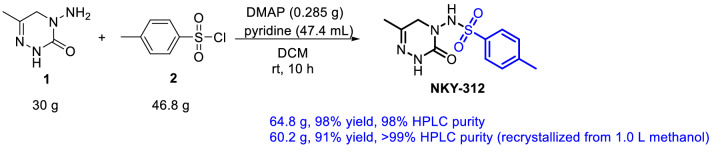
Scaled-up monosulfonylation reaction.

Using the optimized conditions, we carried out a reaction of 30 g of **1** (Fig. 5) and obtained 64.8 g of NKY-312 (98% yield) with 98% HPLC purity; recrystallization from 1 L methanol afforded 60.2 g of NKY-312 (91% yield) with > 99% HPLC purity (determined by an external standard method; details are provided in Figure S3).

### Control experiment

**Figure 6:**
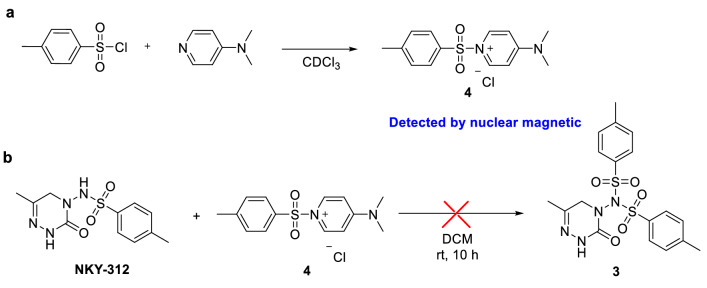
Control experiment. (**a**) *p*-toluene sulfonyl chloride reacted with DMAP in deuterium chloroform. (**b**) the salt **4** did not react with the monosulfonylation product.

To study the mechanism of this DMAP-catalyzed monosulfonylation reaction, some control experiments were performed. When *p*-toluene sulfonyl chloride reacted with DMAP (The mole ratio is 1:1) in deuterium chloroform at room temperature, 1-tosyl-4-dimethylaminopyridinium chloride can be generated in situ (Fig. 6a). We synthesized 1-tosyl-4-dimethylaminopyridinium chloride according to a literature procedure^31^, and we confirmed its structure by means of NMR spectroscopy (see the supporting information for details). When the synthesized salt was allowed to react with NKY-312 under the standard conditions, no bissulfonylated by-product was obtained, which indicates that the salt did not react with the monosulfonylation product (Fig. 6b).

### Proposed reaction mechanism

**Figure 7:**
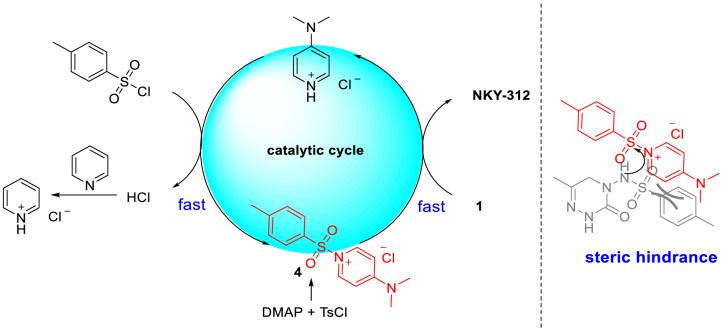
Proposed reaction mechanism.

On the basis of control experiments and literature precedents^31^, we propose the mechanism outlined in Fig. 7. First, *p*-toluene sulfonyl chloride reacted with DMAP to form **4**, and second, the nucleophilic substrate **1** attacks **4** to release the product NKY-312 and generates DMAP∙HCl (which is a very fast process). With the help of pyridine, DMAP∙HCl reacts with *p*-toluene sulfonyl chloride to regenerate **4** (fast reaction) and complete the catalytic cycle. The steric hindrance prevents **4** from further reacting with NKY-312 to form bissulfonylated by-products **3**.

### Field trials

After the completion of process optimization and sample preparation, NKY-312 was employed to evaluate its insecticidal activities against rice planthopper and wheat aphid in field trials using pymetrozine and imidacloprid as controls respectively. The results exhibited that NKY-312 showed the same efficacy as the controls. Tables 3 and 4 showed part of the results of the field trials.

**Table 3:** Results of field trials against rice planthopper of NKY-312 and pymetrozine (Tianjin Institute of Plant Protection, China).

Compd	Effective dose (g/hm^2^)	Average control effect (%)		
		2 days	7 days	10 days
NKY-312	80	74.88	85.30	84.63
Pymetrozine	80	75.92	85.16	84.38

**Table 4:** Results of field trials against wheat aphid of NKY-312 and Imidacloprid (Hunan Chemical Research Institute Co., Ltd, China).

Compd	Effective dose (g/hm^2^)	Average control effect (%)		
		1 day	3 day	7 day
NKY-312	60	62.18	99.82	100
Imidacloprid	60	56.01	99.48	100

In summary, we have developed a process for DMAP-catalyzed monosulfonylation of **1** to obtain insecticide candidate NKY-312. This process, which afforded NKY-312 in 98% yield, with 98% HPLC purity, was highly selective for the monosulfonylation product, did not use pyridine as a solvent, and afforded a higher yield than the previously reported synthesis of this compound. In addition, the process could be used to prepare more than 60 g of NKY-312. After completing the process optimization, we conducted the field trials and toxicity tests. In field experiments, the activities of NKY-312 against rice planthopper and wheat aphid were equal to pymetrozine and imidacloprid respectively. This compound has a very good prospect in commercial development.

## Methods

### General

All reagents were obtained from commercial suppliers and used as received, assuming 100% purity. *N*-(6-Methyl-3-oxo-2,5-dihydro-1,2,4-triazin-4(3*H*)-yl)acetamide (CAS. no. 136738-23-3) was purchased from Chemieliva Pharmaceutical Co. Reaction progress was monitored by thin-layer chromatography (TLC) on silica gel GF254 with UV detection. Melting points (mp) were obtained with an X-4 binocular microscope melting point apparatus and are uncorrected. ^1^H and ^13^C NMR spectra of samples in CDCl_3_ or *d*_6_-DMSO were recorded with a Bruker AV400 spectrometer; tetramethylsilane was used as an internal standard. Chemical shifts (*δ*) are given in parts per million (ppm). Mass spectra were obtained with a Fourier transform ion cyclotron resonance mass spectrometer (ionspec, 7.0 T). HPLC was performed on an Agilent 1260 Infinity II chromatograph with a VP-ODS column (4.6 mm × 250 mm, 5 μm) under the following conditions: mobile phase, 80:20 MeCN/H_2_O; flow rate, 1.0 mL/min; column temperature, 40 °C; UV detection wavelength, 220 nm; detection time, 20 min. An external standard curve method (quantitative method) was used to determine the purity of the final product (NKY-312). Double-recrystallized NKY-312 (HPLC purity > 99.5%) was used as a reference substance.

### Preparation of 4-amino-6-methyl-4,5-dihydro-1,2,4-triazin-3(2*H*)-one (1)

Concentrated HCl (12 mol/L, 36.8 mL, 0.45 mol) was added dropwise to a solution of *N*-(6-methyl-3-oxo-2,5-dihydro-1,2,4-triazin-4(3*H*)-yl)acetamide (50 g, 0.30 mol) in methanol (500 mL) at room temperature, and the resulting mixture was heated at reflux until the reaction was complete, as indicated by TLC (15:1 DCM/methanol). After the reaction solution cooled to room temperature, the pH was adjusted to 7 with 50% sodium NaOH solution, and the solvent was removed by evaporation in vacuo. The residue was taken up in 125 mL of ethanol, and the solvent was again removed in vacuo. Finally, the residue was taken up in 700 mL of acetonitrile, undissolved precipitates (salts) were removed by filtration, and the filtrate was concentrated in vacuo to give **1** (35.0 g, 93%) as a light yellow solid. Mp 120–122 °C. ^1^H NMR (400 MHz, *d*_6_-DMSO) *δ* 9.54 (s, 1H, NH), 4.60 (s, 2H, NH_2_), 3.93 (s, 2H, CH_2_), 1.83 (s, 3H, Me); ^13^C NMR (100 MHz, *d*_6_-DMSO) *δ* 153.6, 144.8, 52.5, 20.3.

### Preparation of NKY-312

Amino-6-methyl-4,5-dihydro-1,2,4-triazin-3(2*H*)-one (**1**; 30 g, 0.234 mol), pyridine (47.4 mL, 2.5 equiv), and DMAP (0.285 g) were dissolved in 225 mL of DCM, and the resulting white suspension was stirred at room temperature for 15 min. Then *p*-toluenesulfonyl chloride (**2**; 46.8 g, 1.05 equiv) dissolved in 195 mL of DCM was added dropwise over the course of about 180 min. About 20 min after the start of the addition of **2**, the reaction mixture gradually became a yellow solution. After the addition was completed, the reaction was allowed to continue for 10 h at room temperature, at which point TLC (60:1 DCM/methanol) indicated that the reaction was complete. Note that a white suspension formed during the course of the 10 h reaction. DCM (360 mL) and dilute HCl (510 mL, 1 mol/L) were added, and the resulting two phases were separated in a separatory funnel. The aqueous phase was extracted with DCM (250 mL × 3), and the combined organic phases were washed with water (600 mL) and saturated brine (600 mL), dried with anhydrous sodium sulfate (about 10 g), and filtered. Evaporation of the filtrate gave NKY-312 (64.8 g, 98% yield, 98% HPLC purity). Recrystallization of the crude product from methanol (1.0 L) afforded 60.2 g of the desired product (91% yield) as a white solid (> 99% HPLC purity, quantitative method; mp 206–208 °C).^1^H NMR (400 MHz, *d*_6_-DMSO) *δ* 10.09 (s, 1H, NH), 9.78 (s, 1H, NH), 7.69 (dd, *J* = 8.0 Hz, 1.2 Hz, 2H, Ar–H), 7.37–7.35 (m, 2H, Ar–H), 4.05 (s, 2H, CH_2_), 2.37 (s, 3H, CH_3_), 1.83 (s, 3H, CH_3_); ^13^C NMR (100 MHz, *d*_6_-DMSO) δ 151.1, 146.0, 143.9, 136.3, 129.7, 128.2, 53.0, 21.4, 20.2. ESI-HRMS (m/z): Calcd. for C_11_H_15_N_4_O_3_S [M + H]^+^ 283.0859; found 283.0863.

### Data for bissulfonylated by-product 3

Dissulfonylated by-product **3** was obtained as a white solid (mp 219–220 °C) by means of recrystallization from a 50:1 mixture of methanol and acetonitrile. ^1^H NMR (400 MHz, *d*_6_-DMSO) *δ* 10.21 (s, 1H, NH), 7.78 (d, *J* = 8.4 Hz, 4H, Ar–H), 7.47 (d, *J* = 8.4 Hz, 4H, Ar–H), 4.20 (s, 2H, CH_2_), 2.44 (s, 6H, CH_3_), 1.87 (s, 3H, CH_3_); ^13^C NMR (100 MHz, *d*_6_-DMSO) *δ* 149.9, 146.6, 146.3, 135.2, 130.2, 129.1, 53.4, 21.6, 20.3. ESI-HRMS (m/z): Calcd. for C_18_H_21_N_4_O_5_S_2_[M + H]^+^ 437.0948; found 437.0947.

### Preparation of 1-tosyl-4-dimethylaminopyridinium chloride(4)

1-Tosyl-4-dimethylaminopyridinium chloride was prepared according to a literature procedure^32^. Briefly, DMAP (767 mg, 6.29 mmol) was dissolved in dry EtOAc (40 mL), and the solution was cooled with an ice-water bath. *p*-Toluenesulfonyl chloride (996 mg, 5.22 mmol) in dry EtOAc (12.5 mL) was added by means of a syringe. The reaction mixture was allowed to warm to room temperature and kept at that temperature for at least 22 h. The product was filtered from the solution, washed thoroughly with diethyl ether (50 mL × 3), and dried in vacuo to afford 1.30 g (79%) of a bright white solid (mp 128–130 °C). The spectral data is consistent with the literature data.

## Supplementary Information

Supplementary Information
