# Supplementary material for: Synthesis process optimization and field trials of insecticide candidate NKY-312
Source: Sci Rep. 2021 Mar 25;11:6895. doi: 10.1038/s41598-021-86475-w (PMC7994830; doi:10.1038/s41598-021-86475-w)
Supplement: Supplementary file 1 — Supplementary Information [file 41598_2021_86475_MOESM1_ESM.pdf]

Supporting Information

Synthesis Process Optimization and Field Trials of Insecticide Candidate NKY-312

Haiqi Wang,[1,2] Hongjian Song[1]*
[1]State Key Laboratory of Elemento-Organic Chemistry, College of Chemistry, Nankai University, Tianjin 300071, People's Republic of China
[2] Department of Chemistry, School of Science, Tianjin University, Tianjin 300072, People's Republic of China

Content

General information.....S2
NMR spectra of compound **1**, NKY-312, and **3**.....S3-S5
X-ray structure and data of NKY-312 and **3**.....S6-S12
Liquid chromatographic conditions.....S13
HPLC standard curve.....S13
Liquid chromatogram of NKY-312.....S14-S15
In-situ synthesize 1-tosyl-4-dimethylaminopyridinium chloride.....S16

General Information. All of the reagents were commercially available and used as purchased assuming 100% purity. *N*-(6-methyl-3-oxo-2,5-dihydro-1,2,4-triazin-4(3H)-yl)acetamide (CAS. No. 136738-23-3, purchased from Chemieliva Pharmaceutical Co.). Reaction progress was monitored by thin-layer chromatography (TLC) on silica gel GF254 with ultraviolet (UV) detection. Melting points were obtained using an X-4 binocular microscope melting point (mp) apparatus and are uncorrected. ^{1}H and ^{13}C NMR spectra were recorded utilizing a Bruker AV400 spectrometer with $CDCl_3$ or DMSO-d_6 as solvent and tetramethylsilane as an internal standard. Chemical shifts (δ) are given in parts per million (ppm). Mass spectra were obtained using a Fourier transform ion cyclotron resonance mass spectrometry (FTICRMS) spectrometer (ionspec, 7.0T). HPLC analysis was performed on an Agilent 1260 Infinity II chromatograph with a VP-ODS column (4.6 mm × 250 mm, 5 μm) using the following conditions: mobile phase, MeCN/H_2O (80:20); flow rate, 1.0 mL/min; column temperature, 40 °C; detection, UV 220 nm; detection time, 20 min. An external standard curve method (quantitative method) was used to determine the purities of the final products NKY-312. Double-recrystallized NKY-312 (HPLC purity >99.5%) were used as a reference substance.

NMR Spectra of 1, NKY-312, and 3

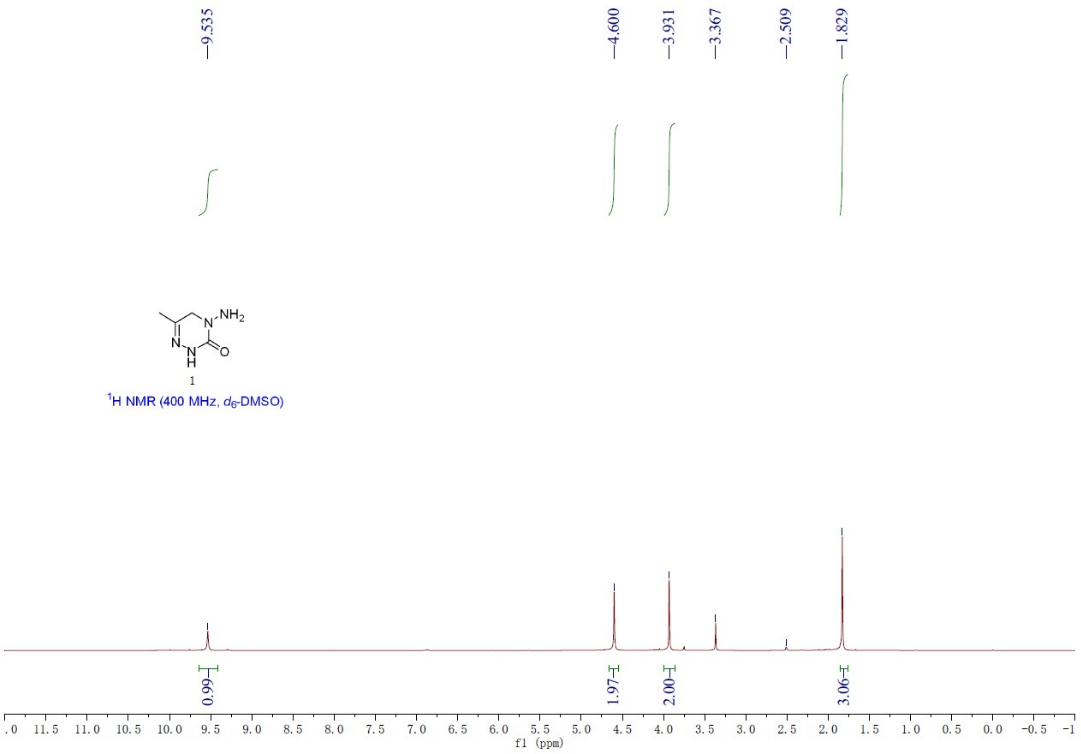

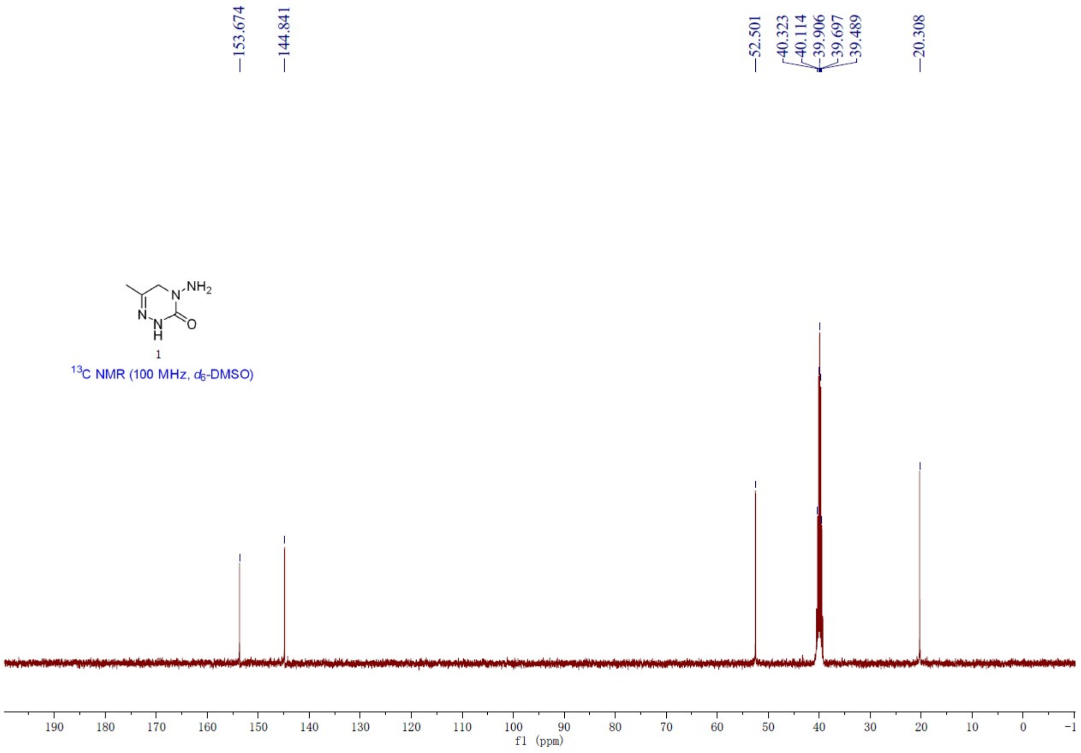

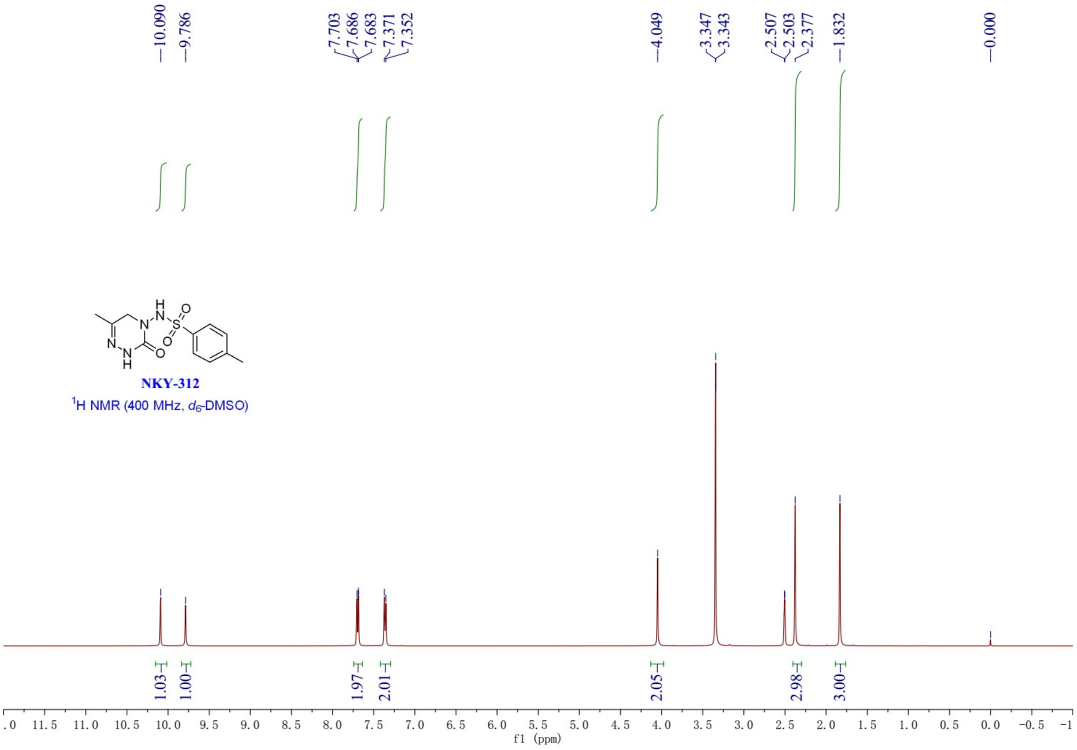
10.090
9.786
7.703
7.686
7.683
7.371
7.352
4.049
3.347
3.343
2.507
2.503
2.377
1.832
0.000
NKY-312
1H NMR (400 MHz, d6-DMSO)
1.03
1.00
1.97
2.01
2.05
2.98
3.00
f1 (ppm)

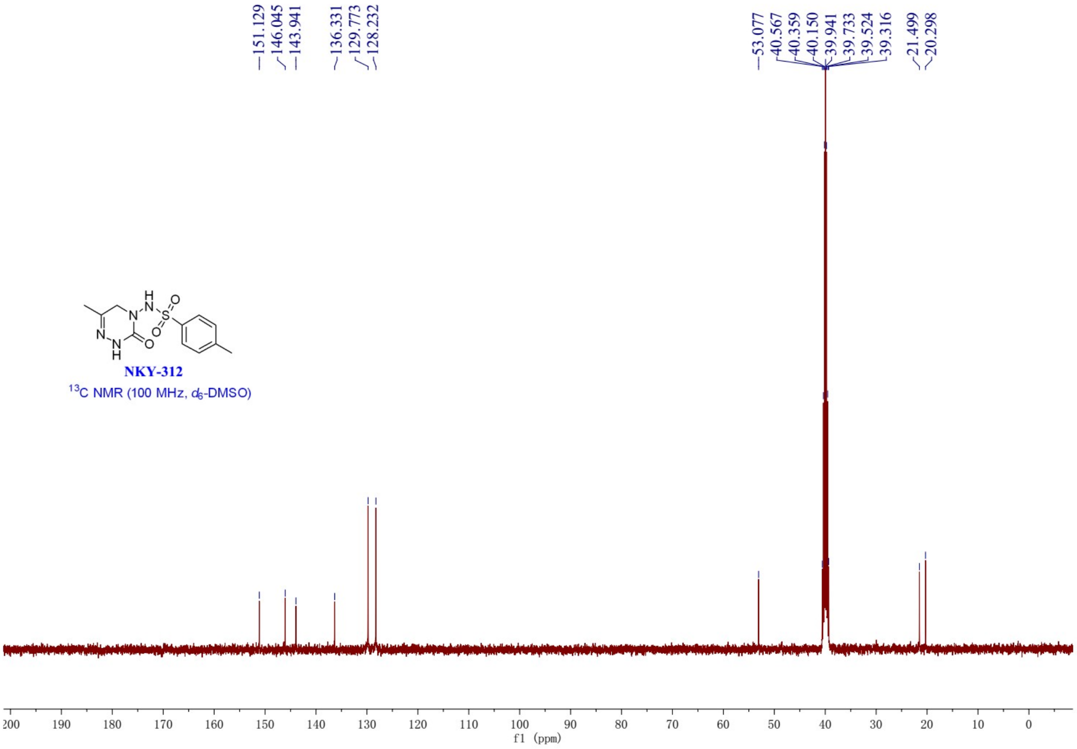
151.129
146.045
143.941
136.331
129.773
128.232
53.077
40.567
40.359
40.150
39.941
39.733
39.524
39.316
21.499
20.298
NKY-312
13C NMR (100 MHz, d6-DMSO)
f1 (ppm)

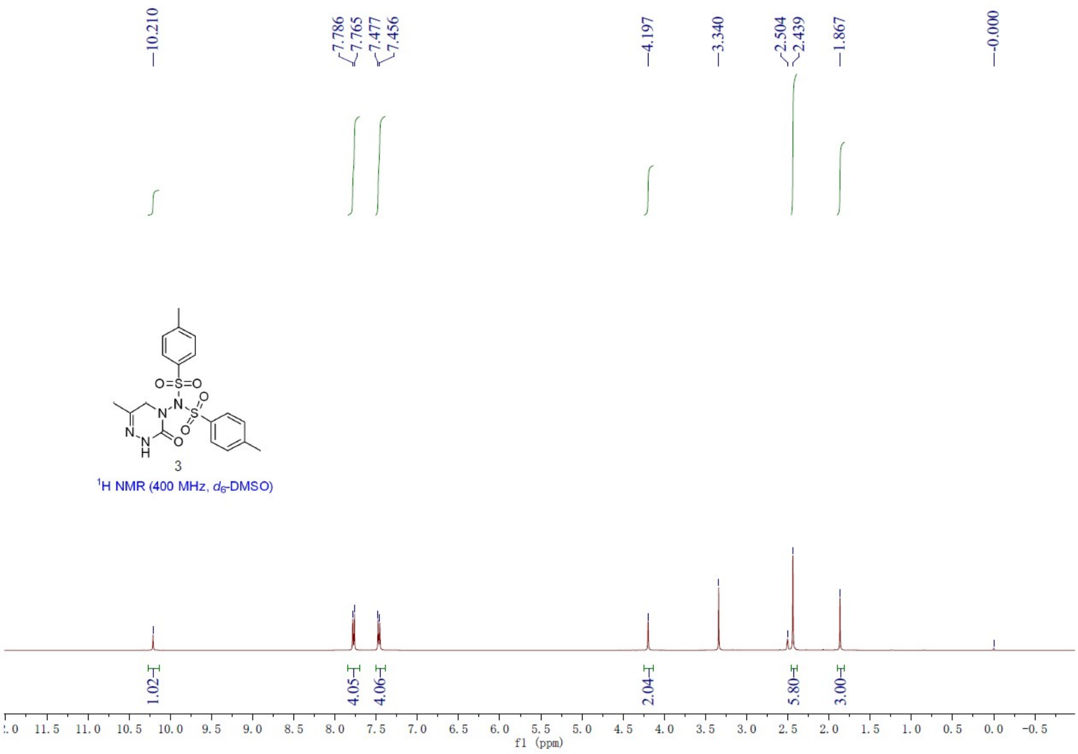
10.210
7.786
7.765
7.477
7.456
4.197
3.340
2.504
2.439
1.867
0.000
O=S=O
N
S
O
O
N
N
H
O
3
1H NMR (400 MHz, d6-DMSO)
1.02
4.05
4.06
2.04
5.80
3.00
11.5 11.0 10.5 10.0 9.5 9.0 8.5 8.0 7.5 7.0 6.5 6.0 5.5 5.0 4.5 4.0 3.5 3.0 2.5 2.0 1.5 1.0 0.5 0.0 -0.5
f1 (ppm)

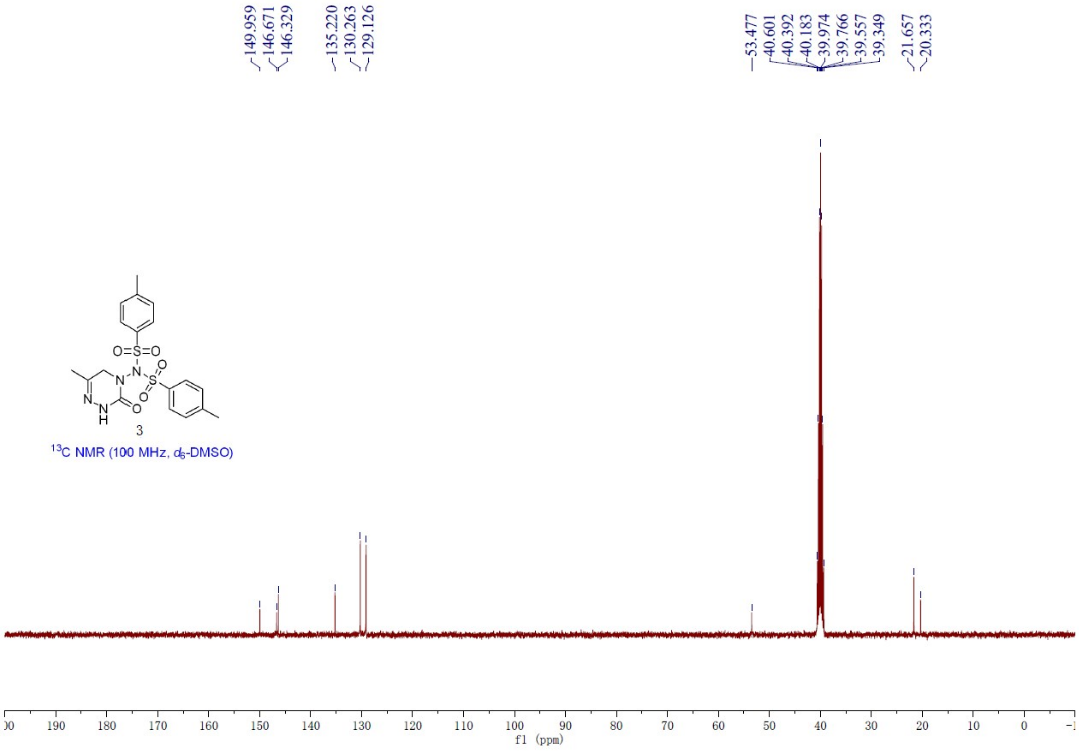
149.959
146.671
146.329
135.220
130.263
129.126
53.477
40.601
40.392
40.183
39.974
39.766
39.557
39.349
21.657
20.333
O=S=O
N
S
O
O
N
N
H
O
3
13C NMR (100 MHz, d6-DMSO)
190 180 170 160 150 140 130 120 110 100 90 80 70 60 50 40 30 20 10 0
f1 (ppm)

Crystal data and structure refinement of NKY-312 and 3

Experimental

Methanol was used as the solvent system; evaporation was used for crystal growth. A suitable crystal of NKY-312 and **3** were selected and collected on a Rigaku Saturn 70 CCD diffractometer. The crystal was kept at 113.15 K during data collection. Using Olex2,[1] the structure was solved with the ShelXS[2] structure solution program using Direct Methods and refined with the ShelXL[3] refinement package using Least Squares minimisation. Crystal data and structure refinement are summarized in Table S1 and Table S2.

Reference

1. Dolomanov, O.V., Bourhis, L.J., Gildea, R.J, Howard, J.A.K. & Puschmann, H. *J. Appl. Cryst.* **2009**, 42, 339-341.
2. Sheldrick, G.M. *Acta Cryst.* **2008**, A64, 11-122.
3. Sheldrick, G.M. *Acta Cryst.* **2015**, C71, 3-8.

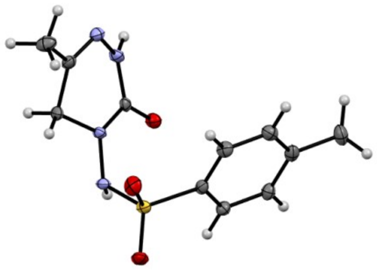

Figure S1. The ORTEP drawing of NKY-312 (Thermal ellipsoids are set at 50% probability level)

Table S1. Crystal data and structure refinement for NKY-312

Identification code	NKY-312
Empirical formula	$C_{11}H_{14}N_4O_3S$
Formula weight	282.32
Temperature/K	113.15
Crystal system	triclinic
Space group	P-1
a/Å	8.1871(8)
b/Å	8.2637(8)
c/Å	10.3359(11)
α/°	86.956(8)
β/°	72.547(9)
γ/°	74.507(9)
Volume/Å^3	642.57(12)
Z	2
ρ_{calc}g/cm^3	1.459
μ/mm^{-1}	0.262
F(000)	296.0
Crystal size/mm^3	0.2 × 0.16 × 0.14
Radiation	MoKα (λ = 0.71073)
2Θ range for data collection/	5.75 to 52.742
Index ranges	-10 ≤ h ≤ 10, -10 ≤ k ≤ 10, -12 ≤ l ≤ 12
Reflections collected	6066
Independent reflections	2521 [R_{int} = 0.0312, R_{sigma} = 0.0335]
Data/restraints/parameters	2521/0/174
Goodness-of-fit on F^2	1.193
Final R indexes [I>=2σ (I)]	R_1 = 0.0633, wR_2 = 0.1636
Final R indexes [all data]	R_1 = 0.0666, wR_2 = 0.1648
Largest diff. peak/hole / e Å^{-3}	0.71/-0.47

No syntax errors found.
Please wait while processing ....

CIF dictionary
Interpreting this report

Datablock: r20200516a

Bond precision:	C-C = 0.0049 A		Wavelength=0.71073
Cell:	a=8.1871(8)	b=8.2637(8)	c=10.3359(11)
	alpha=86.956(8)	beta=72.547(9)	gamma=74.507(9)
Temperature: 113 K			

	Calculated	Reported
Volume	642.57(12)	642.57(12)
Space group	P -1	P -1
Hall group	-P 1	-P 1
Moiety formula	C11 H14 N4 O3 S	C11 H14 N4 O3 S
Sum formula	C11 H14 N4 O3 S	C11 H14 N4 O3 S
Mr	282.32	282.32
Dx,g cm-3	1.459	1.459
Z	2	2
Mu (mm-1)	0.262	0.262
F000	296.0	296.0
F000'	296.37	
h,k,lmax	10,10,12	10,10,12
Nref	2628	2521
Tmin,Tmax	0.951,0.964	0.842,1.000
Tmin'	0.949	

Correction method= # Reported T Limits: Tmin=0.842 Tmax=1.000
AbsCorr = MULTI-SCAN

Data completeness= 0.959 Theta(max)= 26.371

R(reflections)= 0.0633(2353) wR2(reflections)= 0.1648(2521)

S = 1.193 Npar= 174

The following ALERTS were generated. Each ALERT has the format
test-name_ALERT_alert-type_alert-level.
Click on the hyperlinks for more details of the test.

Alert level B

PLAT930_ALERT_2_B FCF-based Twin Law (0 0 1)[-2 0 5] Est.d BASF 0.12 Check

Alert level C

PLAT340_ALERT_3_C Low Bond Precision on C-C Bonds .... 0.00489 Ang.
PLAT906_ALERT_3_C Large K Value in the Analysis of Variance ...... 8.193 Check
PLAT911_ALERT_3_C Missing FCF Refl Between Thmin & STh/L= 0.600 104 Report
PLAT913_ALERT_3_C Missing # of Very Strong Reflections in FCF .... 30 Note
PLAT934_ALERT_3_C Number of (Iobs-Icalc)/Sigma(W) > 10 Outliers .. 1 Check

Alert level G

PLAT007_ALERT_5_G Number of Unrefined Donor-H Atoms .... 2 Report
PLAT019_ALERT_1_G _diffrn_measured_fraction_theta_full/*_max < 1.0 0.995 Report
PLAT870_ALERT_4_G ALERTS Related to Twinning Effects Suppressed .. ! Info
PLAT910_ALERT_3_G Missing # of FCF Reflection(s) Below Theta(Min). 3 Note
PLAT931_ALERT_5_G CIFcalcFCF Twin Law (0 0 1) Est.d BASF 0.12 Check
PLAT933_ALERT_2_G Number of OMIT Records in Embedded .res File ... 9 Note
PLAT941_ALERT_3_G Average HKL Measurement Multiplicity .... 2.4 Low

0 ALERT level A = Most likely a serious problem - resolve or explain
1 ALERT level B = A potentially serious problem, consider carefully
5 ALERT level C = Check. Ensure it is not caused by an omission or oversight
7 ALERT level G = General information/check it is not something unexpected

1 ALERT type 1 CIF construction/syntax error, inconsistent or missing data
2 ALERT type 2 Indicator that the structure model may be wrong or deficient
7 ALERT type 3 Indicator that the structure quality may be low

```
1 ALERT type 4 Improvement, methodology, query or suggestion
2 ALERT type 5 Informative message, check
```

It is advisable to attempt to resolve as many as possible of the alerts in all categories. Often the minor alerts point to easily fixed oversights, errors and omissions in your CIF or refinement strategy, so attention to these fine details can be worthwhile. In order to resolve some of the more serious problems it may be necessary to carry out additional measurements or structure refinements. However, the purpose of your study may justify the reported deviations and the more serious of these should normally be commented upon in the discussion or experimental section of a paper or in the "special_details" fields of the CIF. checkCIF was carefully designed to identify outliers and unusual parameters, but every test has its limitations and alerts that are not important in a particular case may appear. Conversely, the absence of alerts does not guarantee there are no aspects of the results needing attention. It is up to the individual to critically assess their own results and, if necessary, seek expert advice.

Publication of your CIF in IUCr journals

A basic structural check has been run on your CIF. These basic checks will be run on all CIFs submitted for publication in IUCr journals (*Acta Crystallographica*, *Journal of Applied Crystallography*, *Journal of Synchrotron Radiation*); however, if you intend to submit to *Acta Crystallographica Section C* or *E* or *IUCrData*, you should make sure that full publication checks are run on the final version of your CIF prior to submission.

Publication of your CIF in other journals

Please refer to the *Notes for Authors* of the relevant journal for any special instructions relating to CIF submission.

PLATON version of 05/12/2020; check.def file version of 05/12/2020

Datablock r20200516a - ellipsoid plot

Download CIF editor (publCIF) from the IUCr
Download CIF editor (enCIFer) from the CCDC
Test a new CIF entry

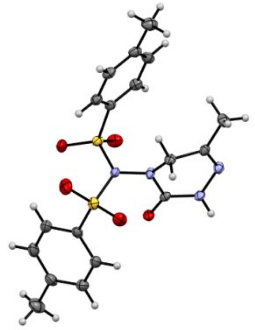

Figure S2. The ORTEP drawing of **7** (Thermal ellipsoids are set at 50% probability level)

Table S2. Crystal data and structure refinement for **3**

Identification code	**3**
Empirical formula	$C_{18}H_{20}N_4O_5S_2$
Formula weight	436.50
Temperature/K	113.15
Crystal system	monoclinic
Space group	$P2_1/n$
a/Å	9.2736(6)
b/Å	10.6892(3)
c/Å	20.829(2)
α/°	90
β/°	100.928(10)
γ/°	90
Volume/Å^3	2027.3(3)
Z	4
ρ_{calc}g/cm^3	1.430
μ/mm^{-1}	0.301
F(000)	912.0
Crystal size/mm^3	0.14 × 0.12 × 0.1
Radiation	MoKα (λ = 0.71073)
2Θ range for data collection/	6.474 to 52.736
Index ranges	-11 ≤ h ≤ 11, -13 ≤ k ≤ 13, -26 ≤ l ≤ 24
Reflections collected	17057
Independent reflections	4112 [R_{int} = 0.0425, R_{sigma} = 0.0326]
Data/restraints/parameters	4112/0/265
Goodness-of-fit on F^2	1.039
Final R indexes [I>=2σ (I)]	R_1 = 0.0367, wR_2 = 0.0934
Final R indexes [all data]	R_1 = 0.0445, wR_2 = 0.0982
Largest diff. peak/hole / e Å^{-3}	0.48/-0.45

No syntax errors found.
Please wait while processing ....

CIF dictionary
Interpreting this report

Datablock: r20200515a

Bond precision:	C-C = 0.0027 A		Wavelength=0.71073
Cell:	a=9.2736(6)	b=10.6892(3)	c=20.829(2)
	alpha=90	beta=100.928(10)	gamma=90
Temperature: 113 K			

	Calculated	Reported
Volume	2027.3(3)	2027.3(3)
Space group	P 21/n	P 1 21/n 1
Hall group	-P 2yn	-P 2yn
Moiety formula	C18 H20 N4 O5 S2	C18 H20 N4 O5 S2
Sum formula	C18 H20 N4 O5 S2	C18 H20 N4 O5 S2
Mr	436.50	436.50
Dx,g cm-3	1.430	1.430
Z	4	4
Mu (mm-1)	0.301	0.301
F000	912.0	912.0
F000'	913.38	
h,k,lmax	11,13,26	11,13,26
Nref	4157	4112
Tmin,Tmax	0.959,0.970	0.438,1.000
Tmin'	0.959	

Correction method= # Reported T Limits: Tmin=0.438 Tmax=1.000
AbsCorr = MULTI-SCAN

Data completeness= 0.989 Theta(max)= 26.368

R(reflections)= 0.0367(3586) wR2(reflections)= 0.0982(4112)

S = 1.039 Npar= 265

The following ALERTS were generated. Each ALERT has the format
test-name_ALERT_alert-type_alert-level.
Click on the hyperlinks for more details of the test.

Alert level C

PLAT906_ALERT_3_C Large K Value in the Analysis of Variance ...... 2.098 Check
PLAT910_ALERT_3_C Missing # of FCF Reflection(s) Below Theta(Min). 7 Note
PLAT911_ALERT_3_C Missing FCF Refl Between Thmin & STh/L= 0.600 36 Report
PLAT913_ALERT_3_C Missing # of Very Strong Reflections in FCF .... 21 Note
PLAT977_ALERT_2_C Check Negative Difference Density on H3 -0.33 eA-3

Alert level G

PLAT007_ALERT_5_G Number of Unrefined Donor-H Atoms .... 1 Report
PLAT380_ALERT_4_G Incorrectly? Oriented X(sp2)-Methyl Moiety ..... C18 Check
PLAT912_ALERT_4_G Missing # of FCF Reflections Above STh/L= 0.600 3 Note
PLAT933_ALERT_2_G Number of OMIT Records in Embedded .res File ... 13 Note
PLAT941_ALERT_3_G Average HKL Measurement Multiplicity .... 4.1 Low
PLAT978_ALERT_2_G Number C-C Bonds with Positive Residual Density. 11 Info

0 ALERT level A = Most likely a serious problem - resolve or explain
0 ALERT level B = A potentially serious problem, consider carefully
5 ALERT level C = Check. Ensure it is not caused by an omission or oversight
6 ALERT level G = General information/check it is not something unexpected

0 ALERT type 1 CIF construction/syntax error, inconsistent or missing data
3 ALERT type 2 Indicator that the structure model may be wrong or deficient
5 ALERT type 3 Indicator that the structure quality may be low
2 ALERT type 4 Improvement, methodology, query or suggestion
1 ALERT type 5 Informative message, check

It is advisable to attempt to resolve as many as possible of the alerts in all categories. Often the minor alerts point to easily fixed oversights, errors and omissions in your CIF or refinement strategy, so attention to these fine details can be worthwhile. In order to resolve some of the more serious problems it may be necessary to carry out additional measurements or structure refinements. However, the purpose of your study may justify the reported deviations and the more serious of these should normally be commented upon in the discussion or experimental section of a paper or in the "special_details" fields of the CIF. checkCIF was carefully designed to identify outliers and unusual parameters, but every test has its limitations and alerts that are not important in a particular case may appear. Conversely, the absence of alerts does not guarantee there are no aspects of the results needing attention. It is up to the individual to critically assess their own results and, if necessary, seek expert advice.

Publication of your CIF in IUCr journals

A basic structural check has been run on your CIF. These basic checks will be run on all CIFs submitted for publication in IUCr journals (*Acta Crystallographica*, *Journal of Applied Crystallography*, *Journal of Synchrotron Radiation*); however, if you intend to submit to *Acta Crystallographica Section C* or *E* or *IUCrData*, you should make sure that full publication checks are run on the final version of your CIF prior to submission.

Publication of your CIF in other journals

Please refer to the *Notes for Authors* of the relevant journal for any special instructions relating to CIF submission.

PLATON version of 05/12/2020; check.def file version of 05/12/2020

Datablock r20200515a - ellipsoid plot

Download CIF editor (publCIF) from the IUCr
Download CIF editor (enCIFer) from the CCDC
Test a new CIF entry

Table S3. Liquid chromatographic condition

Instrument	Agilent 1260
Chromatographic column	SHIMADZU-GL Wonda C act ODS-2 chromatographic column (250mm × 4.6 um)
Mobile phase	CH_3CN:H_2O = 80 : 20
Flow rate	1 mL/min
UV detection wavelength	254 nm
Column temperature	25 °C

HPLC standard curve

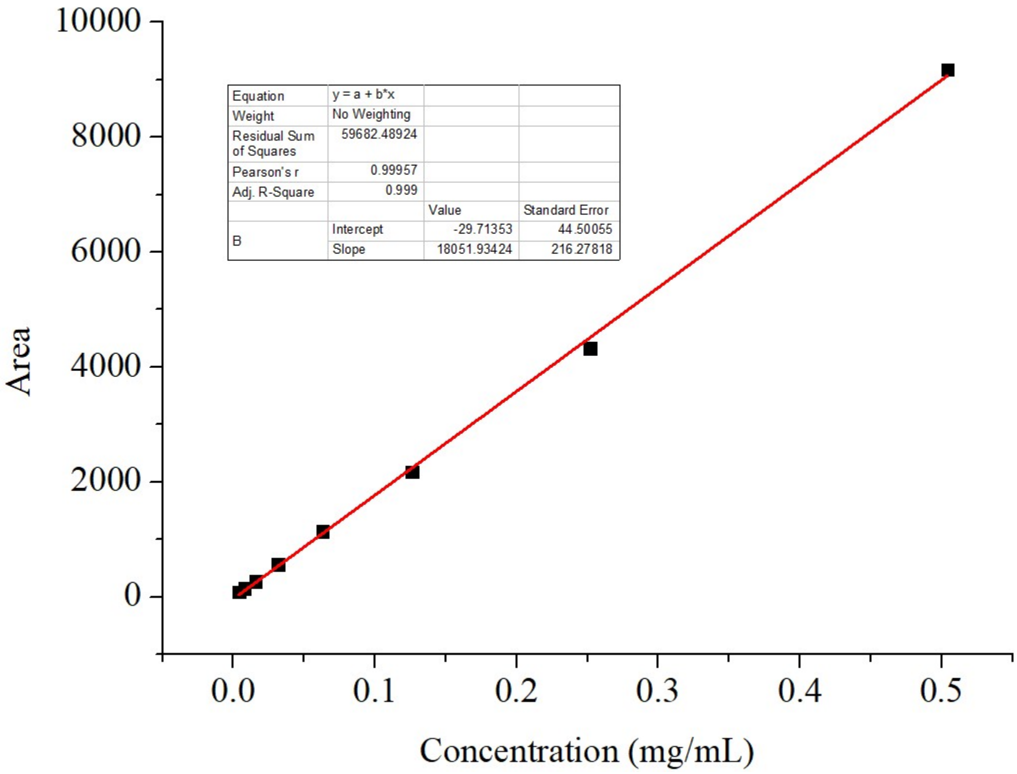

Figure S3. Standard curve

Liquid chromatogram of NKY-312

===

采集操作者　　　：系统
样品操作者　　　：系统
采集仪器　　　　：II　　　　　　　　　　　　位置：　1
进样日期　　　　：2020/7/1 16:11:23　　　　进样次数：　1
　　　　　　　　　　　　　　　　　　　　　　进样量：手动
采集方法　　　　：D:\LC USER\XUWENTAO\NK-0238\20200615\ODS2 CH3CN 80-H2O(NH3.H2O) 20.M
上次更改　　　　：2020/7/1 16:21:11 :　系统
　　　　　　　　　（调用后修改）
分析方法　　　　：D:\LC USER\XUWENTAO\NK-0238\20200615\ODS2 CH3OH 80-H2O 20.M
上次更改　　　　：2020/7/13 10:57:37 :　系统

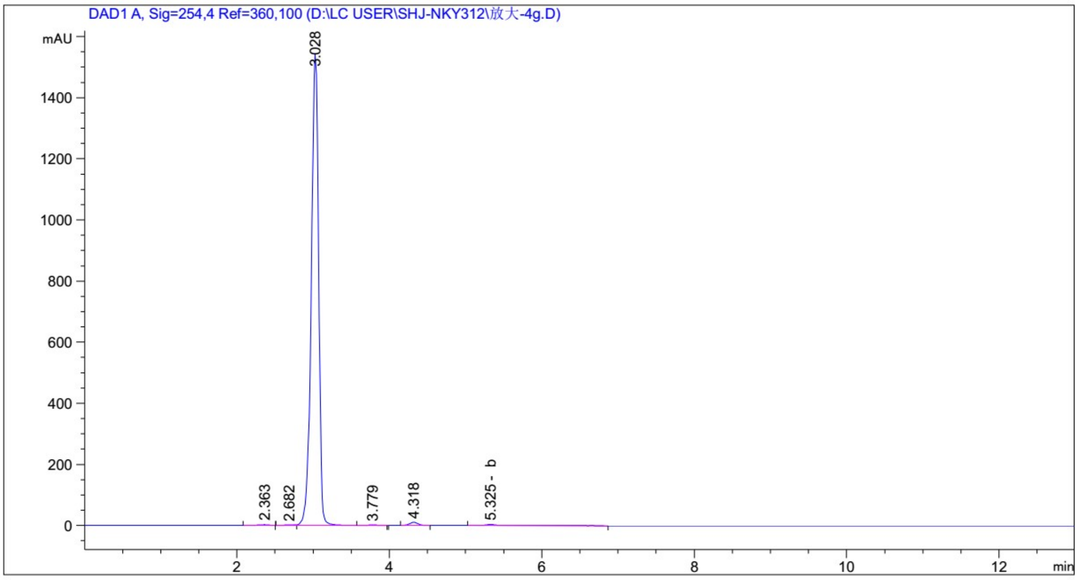

===

面积百分比报告

===

排序　　　　　　　：　信号
校正数据修改时间　：　2019年3月15日15:23:27
乘积因子　　　　　：　1.0000
稀释因子　　　　　：　1.0000
内标中不使用乘积因子和稀释因子

信号 1: DAD1 A, Sig=254,4 Ref=360,100

峰 #	保留时间 [min]	类型	峰宽 [min]	峰面积 [mAU*s]	峰面积 %	名称
1	2.363	BB	0.1413	19.85945	0.1899	?
2	2.682	BV E	0.1027	9.96460	0.0953	?
3	3.028	VV R	0.1018	1.02898e4	98.4029	?
4	3.779	VB E	0.1036	14.06410	0.1345	?

Figure S4. Liquid chromatogram of **NKY-312** (60 gram)

===
采集操作者　　：系统
样品操作者　　：系统
采集仪器　　　：II　　　　　　　　　　　　　位置：　1
进样日期　　　：2020/6/8 16:20:08　　　　　　进样次数：　1
　　　　　　　　　　　　　　　　　　　　　　　进样量：手动
采集方法　　　：D:\LC USER\LIUYUXIU\METHOD\VP-ODS CH3CN 80-H2O 20.M
上次更改　　　：2020/6/8 12:35:09 ：　系统
　　　　　　　　（调用后修改）
分析方法　　　：D:\LC USER\XUWENTAO\NK-0238\20200615\ODS2 CH3OH 80-H2O 20.M
上次更改　　　：2020/7/13 10:57:37 ：　系统
附加信息：峰被手动积分

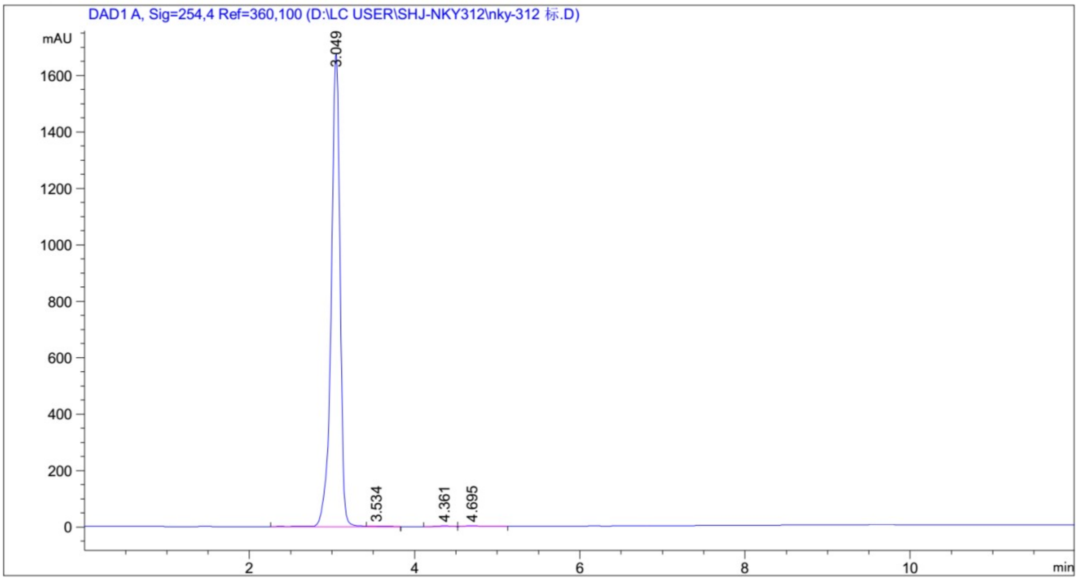

===
面积百分比报告
===

排序　　　　　　　：　　信号
校正数据修改时间　：　　2019年3月15日15:23:27
乘积因子　　　　　：　　1.0000
稀释因子　　　　　：　　1.0000
内标中不使用乘积因子和稀释因子

信号 1: DAD1 A, Sig=254,4 Ref=360,100

峰 #	保留时间 [min]	类型	峰宽 [min]	峰面积 [mAU*s]	峰面积 %	名称
1	3.049	VV R	0.1132	1.23343e4	99.4973	?
2	3.534	VB E	0.1454	10.50727	0.0848	?
3	4.361	BV	0.1422	21.43531	0.1729	?

Figure S5. Liquid chromatogram of **NKY-312** (Recrystallized)

In-situ synthesize 1-tosyl-4-dimethylaminopyridinium chloride

p-Toluene sulfonyl chloride reacted with DMAP (The mole ratio is 1:1) in deuterium chloroform at room temperature, and the reaction solution was taken for nuclear magnetic test.

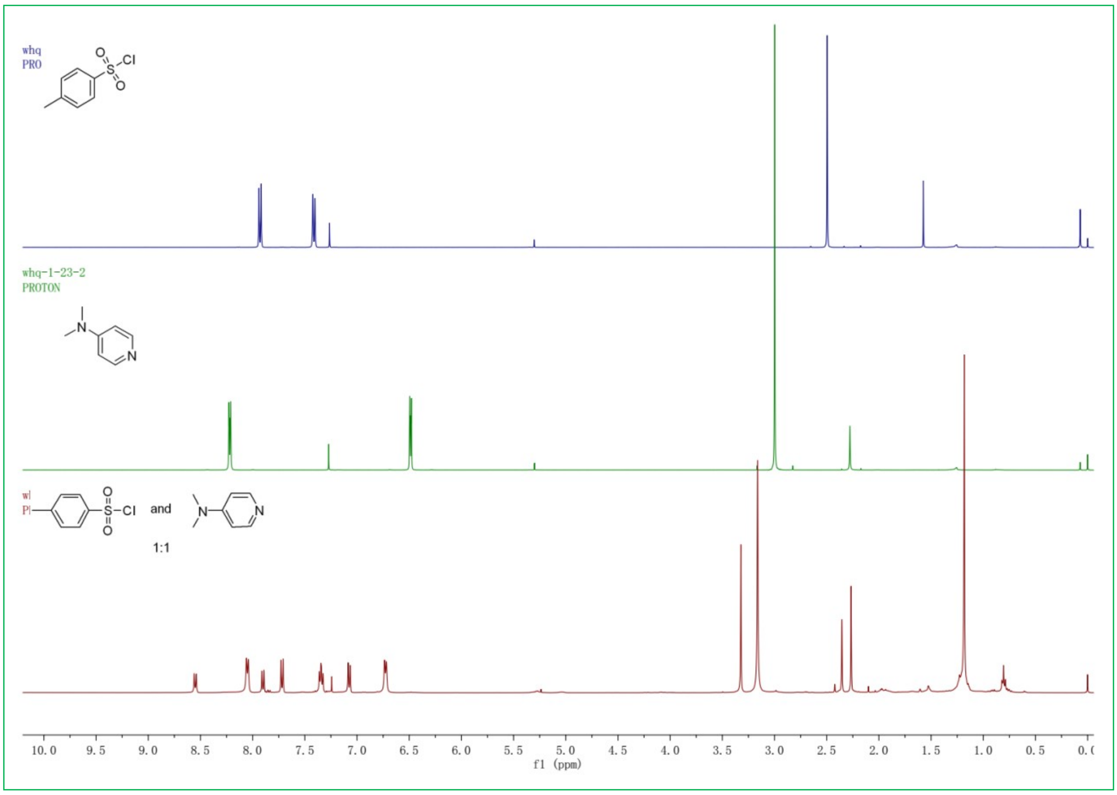

Figure S6. ^{1}H NMR data of the in-situ synthesize 1-tosyl-4-dimethylamino pyridinium chloride
